# Meta-analyses of genome wide association studies in lines of laying hens divergently selected for feather pecking using imputed sequence level genotypes

**DOI:** 10.1186/s12863-020-00920-9

**Published:** 2020-10-01

**Authors:** Clemens Falker-Gieske, Hanna Iffland, Siegfried Preuß, Werner Bessei, Cord Drögemüller, Jörn Bennewitz, Jens Tetens

**Affiliations:** 1 Department of Animal Sciences, Georg-August-University, Burckhardtweg 2, 37077 Göttingen, Germany; 2 Institute of Animal Science, University of Hohenheim, Garbenstr. 17, 70599 Stuttgart, Germany; 3 Institute of Genetics, Vetsuisse Faculty, University of Bern, Bremgartenstr. 109a, 3001 Bern, Switzerland; 4 Center for Integrated Breeding Research, Georg-August-University, Albrecht-Thaer-Weg 3, 37075 Göttingen, Germany

**Keywords:** Feather pecking, Whole genome sequencing, Imputation, Meta-analysis, Genome wide association study, Chicken

## Abstract

**Background:**

Feather pecking (FP) is damaging behavior in laying hens leading to global economic losses in the layer industry and massive impairments of animal welfare. The objective of the study was to discover genetic variants and affected genes that lead to FP behavior. To achieve that we imputed low-density genotypes from two different populations of layers divergently selected for FP to sequence level by performing whole genome sequencing on founder and half-sib individuals. In order to decipher the genetic structure of FP, genome wide association studies and meta-analyses of two resource populations were carried out by focusing on the traits ‘feather pecks delivered’ (FPD) and the ‘posterior probability of a hen to belong to the extreme feather pecking subgroup’ (pEFP).

**Results:**

In this meta-analysis, we discovered numerous genes that are affected by polymorphisms significantly associated with the trait FPD. Among them *SPATS2L*, *ZEB2*, *KCHN8*, and *MRPL13* which have been previously connected to psychiatric disorders with the latter two being responsive to nicotine treatment. Gene set enrichment analysis revealed that phosphatidylinositol signaling is affected by genes identified in the GWAS and that the Golgi apparatus as well as brain structure may be involved in the development of a FP phenotype. Further, we were able to validate a previously discovered QTL for the trait pEFP on GGA1, which contains variants affecting *NIPA1*, *KIAA1211L*, *AFF3*, and *TSGA10*.

**Conclusions:**

We provide evidence for the involvement of numerous genes in the propensity to exhibit FP behavior that could aid in the selection against this unwanted trait. Furthermore, we identified variants that are involved in phosphatidylinositol signaling, Golgi metabolism and cell structure and therefore propose changes in brain structure to be an influential factor in FP, as already described in human neuropsychiatric disorders.

## Background

Feather pecking (FP) is a worldwide problem in layers substantially impairing animal welfare and causing economic losses. The propensity to show this behavior is a complex trait affected by various environmental factors and numerous genes. Heritability estimates of around 0.15 were reported [1–3] indicating the possibility to select against this unwanted behavior. Despite decades of intensive research, the causative mechanisms underlying FP are still not completely understood. The mapping of causative genes for FP is challenging because the recording of individual phenotypes requires labor-intensive behavioral observations. Thus, to address this problem, feather condition can be used instead and considered as a social interaction trait affected by FP behavior of conspecifics [4].

It is, however, unpredictable in commercial layer populations, whether and when FP will occur. Thus, most studies have been conducted in layer lines divergently selected for FP behavior [5]. Genomic regions putatively affecting FP were identified by mapping selection signatures within these lines [2, 6], as well as by classical quantitative trait loci (QTL) mapping [7] and genome wide associations study (GWAS) approaches in resource populations established from the lines such as F2 populations [8]. Also, candidate gene approaches [9] have been adopted. In summary, mapping studies identified loci connecting FP to dopaminergic [9], GABAergic [6] and serotonergic signaling [2, 4, 9] as well as to immune function [4]. Since these studies were based on low-density and medium-density marker panels, no candidate mutations were pinpointed. Here we present an approach in which we performed whole-genome sequencing (WGS) on selected animals, in order to impute low-level chip genotype data to sequencing level. We have already successfully applied this strategy in a porcine F2 design [10]. In the present study, we combined the results of GWAS from two resource populations: an F2 design and one half-sib (HS) population divergently selected for feather pecking behavior. This type of meta-analysis was already established in pigs [11] and applied in an FP analysis, which in addition included selection signatures [8]. The same strategy enabled us to identify 15 genome-wide significant variants associated with FP behavior in the present study. The variant with the lowest *p*-value (rs734668878) is an intron-variant of the *SPATS2L* gene and is located on GGA7. *SPATS2L* has been associated with schizophrenia in numerous studies [12–17].

## Results

### Whole-genome sequencing, variant calling, and imputation accuracy

An average of 250,771,841 (SD = 154,322,025; MIN = 123,212,910; MAX = 815,729,265) sequencing reads per sample were aligned to the reference genome with an average mapping efficiency of 98.39%. The mean coverage of sequenced F0 individuals was 14.42 and of HS individuals 23.49. In total 12,864,421 SNPs were detected, 1,219,711 of which were novel as of dbSNP version Galgal_variation_release-92. With respect to the number of INDELs, 1,561,896 out of 2,142,539 variants were novel. The Ti/Tv of SNPs was 2.403, whereas known SNPs had a Ti/Tv of 2.449 and novel SNPs had a Ti/Tv of 1.965. The coefficients of determination for the imputation accuracy with the HS population were as follows: GGA1, R^2^ = 0.740 (SD = 0.351), GGA7, R^2^ = 0.767 (SD = 0.342). R^2^ values for all tested markers are summarized in Additional File 1.

### Variance explained by markers, GWAS and variant effect prediction

Based on the SNP-chip data, the phenotypic variance explained by all markers (chip-heritability) was estimated as 0.18 (S.E. 0.05) in the F2 and 0.20 (S.E. 0.08) in the HS design, respectively. Using the imputed sequence level data, a GWAS was performed for the trait ‘feather pecks delivered’ (FPD) in each of the designs. After Bonferroni correction, 16 significant variants (*p*-value < 7.421 × 10^− 8^) were detected in the F2 design and 185 significant variants (p-value < 1.158 × 10^− 7^) were detected in the HS design. Variants with -log10(p) > 5 are summarized in Additional File 2 and Manhattan plots are shown in Additional File 3. The p-value thresholds after Bonferroni corrections differ due to different numbers of variants analyzed (6,737,823 variants in the F2 design, 4,316,405 variants in the HS structure). Variant effect prediction (VEP) revealed that the two study designs, F2 and HS, have no genes in common among those that are affected by variants with -log10(p) > 5 (Venn diagram in Additional File 4). No statistically significant signals were detected in the two GWAS for the ‘posterior probability of animals to be extreme feather peckers’ (pEFP) [18] (Manhattan plots in Additional File 3, variants with -log10(p) > 5 in Additional File 2). The F2 and the HS study designs have no affected genes in common among variants with a -log10(p) > 5 (Venn diagram in Additional File 4).

**Fig. 1:**
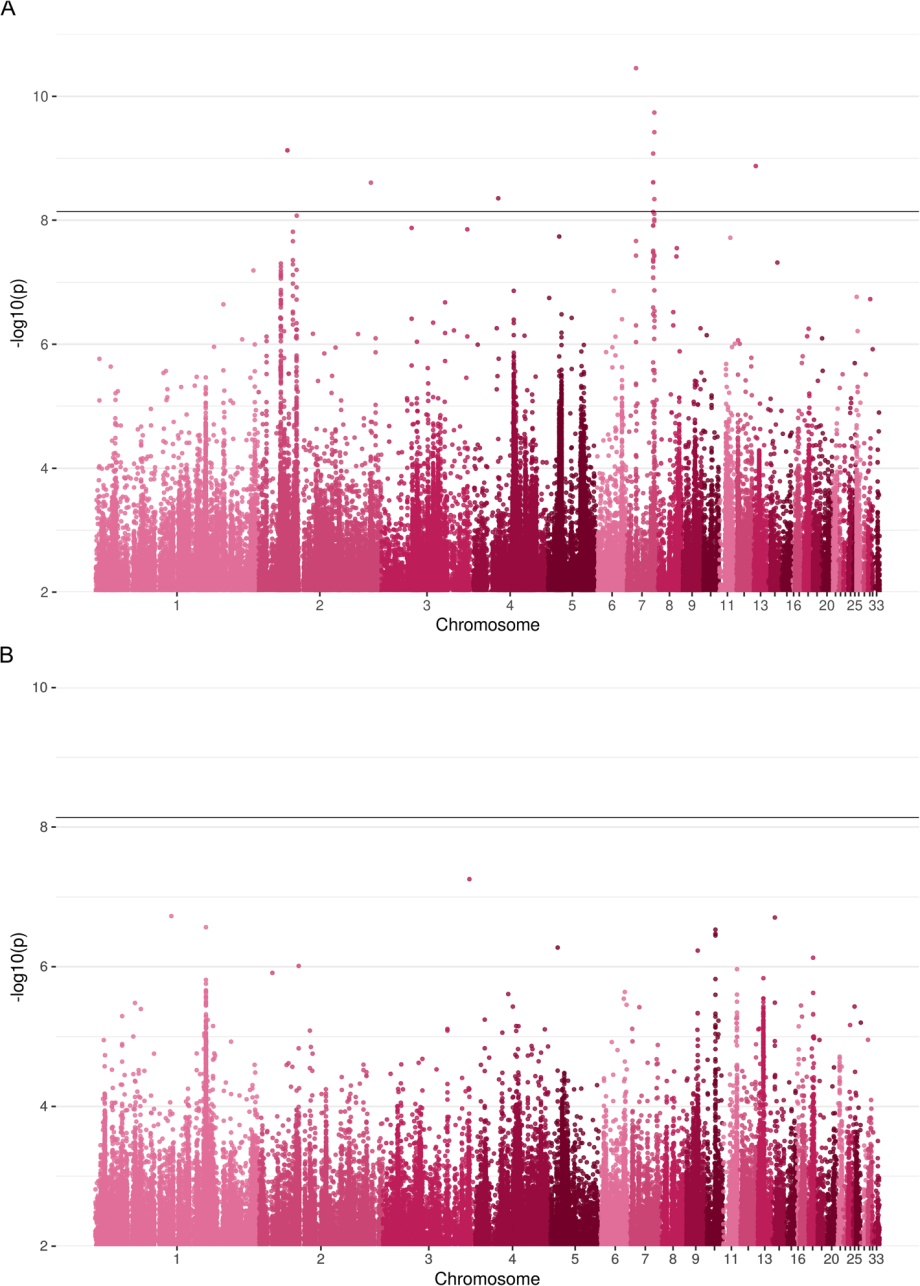
Manhattan plots of the meta-analyses of genome wide associations studies from two resource populations (F2 and HS; half-sib) with the phenotypes (**a**) feather pecks delivered’ (FPD) and (**b**) the ‘posterior probability of a hen to belong to the extreme feather pecking subgroup’ (pEFP)

After performing a meta-analysis of the two FPD data sets, we identified 15 significant variants (*p*-value < 7.248 × 10^− 8^, 6,898,216 variants were included in the analysis), of which 10 variants were also statistically significant in the F2 GWAS and 5 variants in the HS design. A Manhattan plot, which displays the results of the meta-analysis for the phenotype FPD is shown in Fig. 1a and variants with -log10(p) > 5 are summarized in Additional File 2. In the F2 design, the proportion of phenotypic variance jointly explained by all genome-wide significantly associated markers was estimated as 0.08 (S.E. 0.04), while the proportion explained by the QTL region in the HS design was estimated as 0.07 (S.E. 0.05). The variants with significant *p*-values are listed in Table 1. Notably, the meta-analysis of the trait FPD led to the discovery of 111 affected genes, that were not above a -log10(p) threshold of 5 in the two single GWAS (Additional File 5). Only one of those genes, *UNC50*, has been previously associated with FP [19].

**Table 1:** The variants with significant p-values from the meta-analysis of genome wide associations studies from two resource populations (F2 and HS; half-sib) with the trait ‘feather pecks delivered’ (FPD)

Chr	Position (bp)	ID	p-value	Variant effect	Symbol
7	11,147,641	rs734668878	3.546E-11	intron	*SPATS2L*
7	33,398,007	rs13600324	1.828E-10	intron	*ZEB2*
7	33,397,356	rs315633354	3.791E-10	intron	*ZEB2*
2	35,421,866	rs794115893	7.451E-10	intergenic	*KCNH8*
2	35,420,998	2_35,420,998	7.451E-10	intergenic	*KCNH8*
2	35,422,624	rs732446499	7.451E-10	intergenic	*KCNH8*
2	35,422,366	2_35,422,366	7.451E-10	intergenic	*KCNH8*
2	35,417,854	rs733161790	7.451E-10	intergenic	*KCNH8*
7	32,004,828	rs317095182	8.392E-10	intron	*LRP1B*
13	3,974,978	rs312579842	1.336E-09	intron	*FGF18*
7	32,009,461	rs737373523	2.448E-09	intron	*LRP1B*
7	32,001,224	7_32,001,224	2.448E-09	intron	*LRP1B*
2	136,907,571	2_136,907,571	2.48E-09	downstream gene	*MRPL13*
4	31,165,304	rs732945108	4.438E-09	downstream gene	*MMAA*
7	33,397,298	rs16611918	4.575E-09	intron	*ZEB2*

No statistically significant variants were found in the meta-analysis of the two pEFP datasets (Fig. 1b), although 16 additional genes were found that are affected by variants with –log10(p) > 5 (Additional File 5). The results of the VEP for variants with -log10(p) > 5 for both phenotypes are summarized in Additional File 6.

However, among variants with –log10(p) > 5 the traits FPD and pEFP datasets have 16 affected genes in common (Table 3). It is noteworthy that the variant with the lowest *p*-value for the phenotype pEFP (rs740633021, *p*-value = 5.533 × 10^− 8^) was also detected in the meta-analysis for FPD (p-value = 1.410 × 10^− 8^). VEP produced two different results for variant rs740633021. It was predicted to be an intron variant of the *NRBP1* gene and an upstream gene variant of ncRNA *LOC107053158*. An intron variant affecting *SPATS2L* (rs734668878, FPD *p*-value = 3.546 × 10^− 11^, pEFP p-value = 3.804 × 10^− 6^) was detected in the meta-analyses of both phenotypes as well.

Positive genetic correlations between FP and performance have been reported [20], which might be regarded as a cause for the frequent occurrence of FP in layers. Thus, we also compared our gene lists (Tables 1, 2 and 3) with gene entries in the ChickenQTLdb (https://www.animalgenome.org/cgi-bin/QTLdb/GG/index) related to QTL for production traits and only identified a single gene. *LPP*, noteworthy one of the genes found in common between FPD and pEFP (Table 3), has been reported to be linked to shank length and diameter [21] as well as Bursa of Fabricius weight [22]. The latter finding might correspond the observation of feather pecking being related to the immune system, but this is speculative and needs to be further investigated.

**Table 2:** The top 20 variants with the lowest *p*-values from the meta-analysis of genome wide associations studies from two resource populations (F2 and HS; half-sib) with the trait ‘posterior probability of a hen to belong to the extreme feather pecking subgroup’ (pEFP)

Chr	Position (bp)	ID	p-value	Variant effect	Symbol
3	104,391,861	rs740633021	5.533E-08	intron / upstream gene	*NRBP1 / LOC107053158* (ncRNA)
1	91,991,270	rs737049739	1.878E-07	intergenic	*EPHA6*
14	2,899,119	rs14070749	1.968E-07	non coding transcript / exon	*LOC101750426* (ncRNA)
1	133,743,444	rs15428397	2.710E-07	intron	*KIAA1211L*
10	11,902,712	rs317563905	2.938E-07	downstream gene	*SH3GL3 / LMINA*
10	11,904,575	rs316617403	3.373E-07	downstream gene / 3 prime UTR	*SH3GL3 / LMINA*
10	11,901,170	rs315794181	3.564E-07	downstream gene / 3 prime UTR	*SH3GL3 / LMINA*
5	8,789,444	rs312755711	5.313E-07	intron, non coding transcript	*LOC107053374* (ncRNA)
9	14,663,252	rs14674949	5.865E-07	intron	*LPP*
18	6,202,927	rs736783194	7.428E-07	intergenic	*C18H17ORF67*
2	48,074,231	2_48,074,231	9.747E-07	intron	*PDE1C*
11	16,848,616	rs732938021	0.000001084	intron	*KLHL36*
2	16,335,664	rs731673476	0.000001226	intron	*MYO3A*
13	8,132,912	rs739578589	0.000001461	intron	*GABRB2*
10	11,904,145	rs313724095	0.000001503	downstream gene / 3 prime UTR	*SH3GL3 / LMINA*
1	133,734,332	rs14889804	0.000001544	intron	*KIAA1211L*
1	133,762,004	rs13624872	0.000001753	intron	*KIAA1211L*
1	133,776,463	rs313923430	0.000002173	intron	*TSGA10*
1	133,733,974	rs1059691759	0.000002254	intron	*KIAA1211L*
6	29,989,563	6_29,989,563	0.000002303	intron	*SHTN1*

**Table 3:** Common genes from the genome wide associations studies meta-analyses that are affected by variants with a -log10(p) > 5 for the phenotypes ‘feather pecks delivered’ (FPD) and the ‘posterior probability of a hen to belong to the extreme feather pecking subgroup’ (pEFP)

*Symbol*	ID (FPD)	p-value (FPD)	ID (pEFP)	p-value (pEFP)
*COIL*	rs739114475	5.613E-07	rs731484806	4.79E-06
*DGKE*	rs739114475	5.613E-07	rs731484806	4.79E-06
*KIAA1211L*	rs314041779	0.000003441	rs15428397	2.71E-07
*LMINA*	rs13544839	0.000006722	rs317563905	2.938E-07
*LOC101750426*	rs312554616	0.000007613	rs14070749	1.968E-07
*LOC101752088*	18_7107365	0.000006267	rs794664310	0.000009907
*LOC107053158*	rs740633021	1.41E-08	rs740633021	5.533E-08
*LPP*	rs314190948	0.000009257	rs14674949	5.865E-07
*NRBP1*	rs740633021	1.41E-08	rs740633021	5.533E-08
*PITPNC1*	18_7107365	0.000006267	rs794664310	0.000009907
*RUFY3*	rs732635903	1.371E-07	4_50027189	0.000007014
*SCPEP1*	rs739114475	5.613E-07	rs731484806	0.000004791
*SH3GL3*	rs13544839	0.000006722	rs317563905	2.938E-07
*SPATS2L*	rs734668878	3.546E-11	rs734668878	0.000003804
*TSGA10*	rs738236556	0.000004947	rs313923430	0.000002173
*UTP3*	4_50027189	4.013E-07	4_50027189	0.000007014

### Functional and pathway analyses

**Fig. 2:**
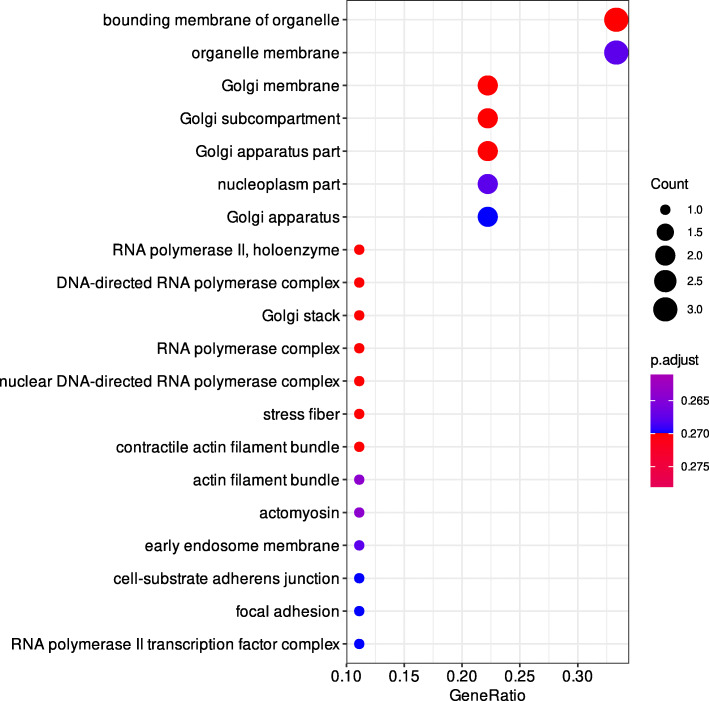
Enriched GO cellular components terms for genes that contain variants with -log10(p) > 5 that were discovered in the meta-analysis of two genome wide associations studies for the trait ‘feather pecks delivered’ (FPD)

Gene cluster analysis with the R package clusterProfiler [23] was performed with genes affected by variants with -log10(p) > 5 from the meta-analyses of GWAS performed on the phenotypes FPD and pEFP. Significant results (q-value > 0.25) that lead to the discovery of categories affected by more than one gene are shown in Table 4. Since the analysis of GO cellular components for the trait FPD led to the discovery of more than 20 terms the results are shown in Fig. 2. The complete clusterProfiler output is summarized in Additional File 7.

**Table 4:** Gene set enrichment analysis of genes affected by variants (−log10(p) > 5) from the genome wide associations studies meta-analyses with the traits ‘feather pecks delivered’ (FPD) and the ‘posterior probability of a hen to belong to the extreme feather pecking subgroup’ (pEFP) with clusterProfiler. Results with q-values > 0.25 and at least two gene symbols are listed

Category	ID	Description	q-value	*Symbols*	Phenotype
KEGG	gga04070	Phosphatidylinositol signaling system	0.000156805	*MTMR7/INPP4B/PLCG2/DGKB/DGKE/ITPR1*	FPD
GO_BP	GO:0006897	endocytosis	0.230358006	*CDH13/SH3GL3*	pEFP
GO_BP	GO:0098657	import into cell	0.230358006	*CDH13/SH3GL3*	pEFP
GO_CC	GO:0005912	adherens junction	0.053757065	*CDH13/LPP*	pEFP
GO_CC	GO:0070161	anchoring junction	0.053757065	*CDH13/LPP*	pEFP
GO_CC	GO:0098805	whole membrane	0.098234855	*CDH13/SH3GL3*	pEFP
GO_CC	GO:0030054	cell junction	0.098234855	*CDH13/LPP*	pEFP

**Fig. 3:**
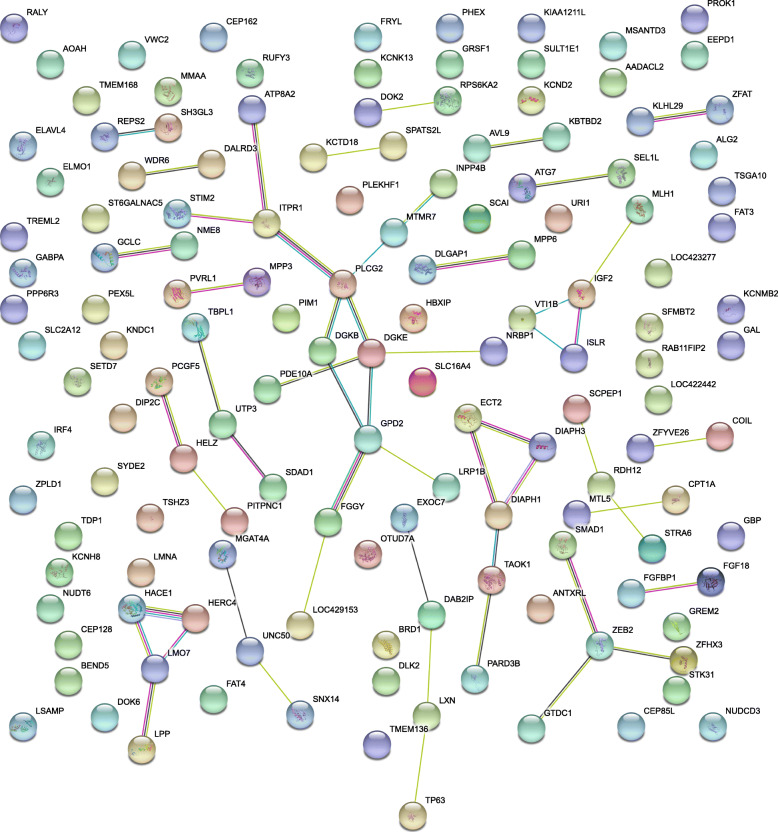
Protein interaction map of genes affected by variants (−log10(p) > 5) from the genome wide associations studies meta-analysis with the trait ‘feather pecks delivered’ (FPD)

Protein interaction network analysis of genes affected by variants with -log10(p) > 5 associated with FPD in the meta-analysis with STRING revealed significantly more interactions than expected (number of edges: 54, expected number of edges: 40, PPI enrichment *p*-value = 0.0197, Fig. 3). Several protein clusters with more than 3 nodes were found: cluster (i) ATP8A2, STIM2, ITPR1, PLCG2, DGKB, DGKE, GPD2, LRP1B, FGGY, and LOC429153; cluster (ii) ECT2, DIAPH3, DIAPH1, TAOK1, and PARD3B; cluster (iii) MLH1, IGF2, VTI1B, and ISLR; cluster (iv) SAMD1, ZEB2, ZFHX3, and GTDC1; cluster (v) HACE1, HERC4, LMO7, and LPP; cluster (vi) EXOC7, DAB2IP, LXN, and TP63. Analysis of the two resource populations separately yielded significantly more interactions than expected in the HS design (number of edges: 17, expected number of edges: 8, PPI enrichment *p*-value = 0.00511) but not in the F2 cross (Additional File 8). The same holds true for the pEFP phenotype.

## Discussion

In the study presented here, we imputed medium-density SNP-chip genotypes of two layer lines divergently selected for FP to sequence level. GWAS and subsequent meta-analyses for the traits ‘*feather pecks delivered’* (FPD) and ‘*posterior probability of belonging to the subgroup of extreme feather peckers’* (pEFP) were performed with both populations. We detected 16 variants that are significantly associated with the trait FPD. The variant with the lowest *p*-value (rs734668878, *p*-value = 3.546 × 10^− 11^) is an intron-variant in the *SPATS2L* gene. Polymorphisms of *SPATS2L* have been linked to hippocampal volume, intelligence, and schizophrenia [12–17], which makes it a promising candidate gene to have an influence on FP behavior. Three statistical significant variants are located in intronic regions of *ZEB2* (lowest *p*-value 1.828 × 10^− 10^), a gene which has been identified to be a risk locus for schizophrenia [24] and to be inducible by nicotine [25]. In proximity to *KCHN8* we discovered five significant variants (lowest p-value 7.451 × 10^− 10^). *KCNH8* was identified in the context of the neuropsychiatric 15q13.3 microdeletion which is associated with several neuropsychiatric disorders including autism, schizophrenia, and attention deficit hyperactivity disorder [26]. Furthermore, we found three intron-variants in *LRP1B* (lowest *p*-value 8.392 × 10^− 10^) to be significantly associated with the trait FPD. *LRP1B* was identified in a GWAS where it was found to be associated with increased ventricular volumes in psychosis [27]. Another promising gene that might influence the propensity to FP is *FGF18*. We found a significantly associated intron-variant (rs312579842, p-value 1.336 × 10^− 9^) in *FGF18*, a gene that has been connected to dyslexia [28]. Furthermore, dysregulation of fibroblast growth factor signaling is currently under discussion to play a role in neurological and psychiatric disorders [29]. Another gene affected by a significantly associated down-stream gene variant is *MRPL13* (2_136,907,571, p-value = 2.480 × 10^− 9^), which was found to be upregulated after nicotine treatment [30] like the already mentioned *ZEB2* gene. We found genes encoding subunits of nicotinic acetylcholine receptors (nAchR), the major receptors for nicotine [31], to be differentially expressed in the brain transcriptome of hens divergently selected for feather pecking [32]. Both findings taken together present strong evidence for the involvement of nAchR in the propensity to FP behavior (already discussed in [32]). Gene cluster analysis with clusterProfiler revealed that genes belonging to the KEGG category “Phosphatidylinositol signaling system” are enriched among genes associated with the trait FPD (Table 4). This discovery is of special interest since the phosphatidylinositol second messenger system is currently under discussion to be involved in the pathophysiology of psychiatric disorders [33]. Furthermore, GO cellular component analysis (Fig. 2) revealed that genes located in the Golgi apparatus and genes involved in cell structure are affected by variants identified in this study. Golgipathies and Golgi fragmentation are currently a matter of discussion for an influential role in neurodegenerative diseases and neurological development [34, 35].

In summary, the polymorphisms affecting the genes discussed above substantially contribute to the explanation of the genetic architecture behind FP behavior. While we estimated a chip heritability that corresponds well with previously published genetic parameters [1–3], the associated variants in this study do explain 7–8% of phenotypic variance for FPD.

Since we discovered more protein interactions among FPD-associated gene products than expected (Fig. 3), we propose that this interaction map provides a basis for downstream functional analysis. The proteins that belong to the largest cluster (cluster (i): ATP8A2, STIM2, ITPR1, PLCG2, DGKB, DGKE, GPD2, LRP1B, FGGY, and LOC429153) confirm the finding from our gene cluster analysis. A large number of these proteins are involved in phosphatidylinositol metabolism but also in other lipid- and membrane-associated processes. The second-largest cluster contains the proteins ECT2, DIAPH3, DIAPH1, TAOK1, and PARD3B, all of which play a role in structural processes. We found another cluster that contains genes, which influence brain development and cell proliferation in the brain (cluster (iv) SAMD1, ZEB2, ZFHX3, and GTDC1). This confirms our findings from the GO cellular component analysis (Fig. 2). Structural anomalies of the brain have been shown to be involved in numerous psychiatric disorders [36]. A comparative analysis of the brain structure of hens that exhibit FP behavior with control animals is necessary to clarify, whether this also holds true for the FP phenotype. Another protein interaction cluster consists of MLH1, VTI1B, ISLR, and IGF2. The *IGF2* gene is especially interesting since it is currently under discussion as a pharmacological target in various human psychiatric conditions (reviewed in [37]). Interaction cluster (v) contains proteins that are involved in ubiquitin transfer (HACE1, HERC4, LMO7, and LPP). The ubiquitin proteasome system is disrupted in neuropsychiatric disorders and has important functions in brain development [38].

In a second meta-analysis, the pEFP as described by Iffland et al. [18] was examined. Extreme feather pecking (EFP) hens are a subgroup of hens that exhibit FP behavior and in case of the F2 cross more than twice as often as hens of the normal feather pecking subgroup. They peck with higher intensities, and peck up to five times more feathers. Interestingly, EFP hens can be found in lines selected for high (HFP) and low feather pecking (LFP) and make up about one-third of the HS population as well as the F2 cross [6, 18]. It seems that extreme pecking and overall propensity to show FP behavior are phenotypically highly correlated, but clearly not identical traits. Thus, overlaps between GWAS results for pEFP and FPD cannot necessarily be expected. Here, significant signals for pEFP were neither detectable in the GWAS of the two resource populations (Additional File 3) nor in the meta-analysis after Bonferroni correction (Fig. 1b). However, we were able to correlate our findings with previous studies by examining variants with -log10(p) > 5. In a previous study partly using the same resource populations, a significant QTL on GGA1 (131,055,669 - 133,345,452 bp) was identified, which was confirmed in the current meta-analysis (Fig. 1b, 131,766,790 - 134,135,880 bp). Based on their mapping results, Iffland et al. [6] reported different GABA-receptor subunits (*GABRA5*, *GABRB3*, and *GABRG3*) as potential candidate genes. That study was carried out using SNP-chip data providing limited mapping resolution. Inspecting the genes in our meta-analysis, using VEP revealed that *NIPA1*, *KIAA1211L*, *AFF3*, and *TSGA10* are genes affected by variants in this region. Copy number variations in *NIPA1* were found in psychiatric disorders [39], *KIAA1211L* was identified in a schizophrenia twin-study [40] and is a candidate gene for opioid abuse [41], and *AFF3* is involved in intellectual disability and cellular migration in the cerebral cortex of mice [42]. The studies mentioned make those genes likely candidates to influence the trait pEFP. However, we did not find evidence for the involvement of the GABA-receptor subunits found by Iffland et al. [6]. In the previous study, medium-density SNP chip data was used and candidate genes were thus identified based on linkage disequilibrium. Our current approach using imputed sequence level genotypes proves superior in this respect as the evaluation of variants within or close to putative candidate genes is possible. Although we were not able to confirm the aforementioned genes related to GABAergic signaling, we were able to detect 52 intron variants, among them two insertions, with -log10(p) > 5 on GGA13 (8,084,380 - 8,135,739 bp), which affect the GABA-receptor subunit *GABRB2* (Additional File 2). For *GABRB2* as well strong evidence exists to be a causative gene for schizophrenia [43–50] and it seems to be involved in opioid addiction [51]. Iffland et al. [6] found the QTL region on GGA1 also associated with FPD, but we were not able to confirm this in our GWAS based on imputed sequence level genotypes. Nevertheless, we found 16 common genes out of 40 (pEFP) and 191 (FPD) affected by polymorphisms with -log10(p) > 5 between the two traits (Table 3) by which we also confirm a small overlap.

Imputation accuracy in the HS population was considerably high with average coefficients of determination between R^2^ = 0.740–0.767. We assume that the imputation accuracy in the F2 population was even higher, due to the usage of pedigree information. However, because of the lack of sequenced individuals we were not able to compute it. Notably, the GWAS for both traits revealed no common significantly associated genes (−log10(p) > 5) between the F2 and HS populations (Venn diagrams in Additional File 4 and gene lists in Additional File 5). It seems possible that the top GWAS hits in the F2 population are based on loci that are fixed or close to fixation in the pure selection lines and are thus not detectable in the HS structure. On the other hand, loci still segregating in one of the lines will be easily detectable if sires are QTL heterozygous. 

## Conclusions

We identified several significant genomic variants associated with the trait FPD, that mostly affect genes that have been previously associated with psychiatric and neurological disorders. Among them *SPATS2L*, *ZEB2*, and *KCHN8*, all of which have been identified in schizophrenia studies [12–17, 24, 26]. Furthermore, the genes *ZEB2* and *MRPL13*, which are affected by significantly associated variants, respond to nicotine treatment [25, 30]. This goes in line with our previous results where we detected differential expression of major receptors for nicotine between HFP and LFP hens [32]. Based on our findings from gene cluster analysis and protein interaction clustering we also propose an involvement of phosphatidylinositol signaling, disturbances of the Golgi apparatus, and structural abnormalities in the brain as possible factors that influence the predisposition for FP behavior. Concerning the trait pEFP we could confirm a QTL on GGA1 that was previously identified [6], which in our study contains variants affecting *NIPA1*, *KIAA1211L*, *AFF3*, and *TSGA10*. All of those genes, except *TSGA10*, have been implicated in neurological conditions [39–42] making them additional targets for genomic selection against FP.

## Methods

### Experimental population

Both experimental populations were derived from White Leghorn lines divergently selected for low and high feather pecking [2, 52]. These lines were created and are maintained at the Hohenheim University and neither commercially obtained nor from a private source. The F2-design was established from the 10th generation of the selection experiment and has been described in detail by Lutz et al. [8]. Briefly, five sires and ten dams of each line were used to generate 10 F1 families; then, 10 F1 sires were used to generate the F2 families. Each sire was mated with eight F1 hens four times by artificial insemination. In total, 960 F2 offspring were produced in four hatches. The half-sib design (HS) was recruited from the 15th generation of the selection experiment by mating six sires per line to hens within the respective line with the aim to produce about 600 animals in total. Animals were phenotyped according to established protocols (see below) and genotyped using an Illumina 60 k SNP chip. We successfully pheno- and genotyped a total of 817 F2 hens and 489 offspring from the HS structure (270 HFP and 219 LFP animals). Chickens were CO_2_-stunned and sacrificed by ventral neck cutting. Blood was drawn directly while the animals were bleeding out.

### Phenotyping

Details on phenotyping and housing conditions can be found in Lutz et al. [8] and Iffland et al. [6]. Briefly, animals were phenotyped at around 32 weeks of age according to established protocols [53, 54] recording the feather pecks delivered. For the pure lines, HFP and LFP animals were kept in a ratio as close to 1:1 as possible. The group size was around 42 animals. Observations were done in sessions of 20 min by seven independent and trained observers. Phenotypic values were standardized to 420 min observation time (referred to as FPD in the manuscript). In addition, we derived the posterior probability of belonging to the subgroup of extreme feather peckers (pEFP) as previously described [18, 55]. This is briefly achieved by fitting a mixture of two negative binomial distributions to FP data [6, 18].

### DNA isolation

DNA was isolated from EDTA conserved blood samples with the Promega Maxwell® 16 and Maxwell® Blood DNA Purification Kits (Kat# AS1010) according to the manufacturer’s protocol. For genotyping with Illumina SNP chips the concentration was adjusted to 50 +/− 10 ng/μl.

### NGS library preparation and sequencing

Illumina PCR-free TruSeq fragment libraries with 350 bp insert size were prepared. We collected on average 128 million 2 × 150 bp paired-end reads on a NovSeq6000 instrument.

### Mapping and variant detection

Mapping and variant calling was performed according to the GATK best practice pipeline using GATK v. 4.0 [56] and chicken genome version GRCg6a (GCF_000002315.5 RefSeq assembly). Briefly, fastq files were converted to bam files with GATK FastqToSam in order to add read group information (Instrument Name, Library ID, run ID, flowcell ID, and flowcell lane). Illumina adapters were marked with GATK MarkIlluminaAdapters. Reads were mapped to the reference genome with bwa-mem version 0.7.12 [57]. Technical artifacts and duplicate reads were marked with GATK MarkDuplicates. Base quality scores were recalibrated with GATK BaseRecalibrator and ApplyBQSR. Variants were called by sample with GATK HaplotypeCaller in ERC mode. The resulting GVCF files were merged with GATK CombineGVCFs and jointly genotyped with GATK GenotypeGVCfs. The resulting raw variants were filtered with GATK VariantRecalibrator and ApplyVQSR. The knownSites dataset was produced by performing a lift-over of Galgal_variation_release-92 (ensemble) from genome Version Gallus_gallus-5.0 (ensemble) to GRCg6a with GATK LiftoverVcf. The chain file for the lift-over was created with flo (https://github.com/wurmlab/flo) with both genomes as input files. The success rate of the dbSNP liftover was 99.35%. Base quality score recalibration was performed with the aforementioned dbSNP version as the knownSites dataset. Truth datasets used for Variant Quality Score Recalibration (VQSR) were as follows. SNPs: Illumina chicken 60 k BeadChip and 600 k Affymetrix Axiom HD chicken genotyping array. INDELs: High confidence fraction (filter settings: QD 30.0, FS 200.0, ReadPosRankSum 20.0) of a variant calling performed on a dataset of 50 laying hens (25 brown layers and 25 white layers) that were already described [58]. Training dataset for SNP VQSR was also a high confidence fraction of the dataset by Ni et al. (filter settings: QD 30.0, FS 60.0, MQ 40.0, MQRankSum 12.5, ReadPosRankSum 8.0, SOR 3.0). A truth sensitivity of 99.0 was chosen for SNPs and INDELs. The known dataset for SNP and INDEL VQSR was the dbSNP database mentioned above. Since SNPs were filtered with two truth datasets, a Ti/Tv free recalibration according to the GATK best practice guidelines was applied to the data. Variant IDs for novel variants were generated automatically by combining the chromosome number with the variant position in bp.

### Haplotype construction and imputation

**Fig. 4:**
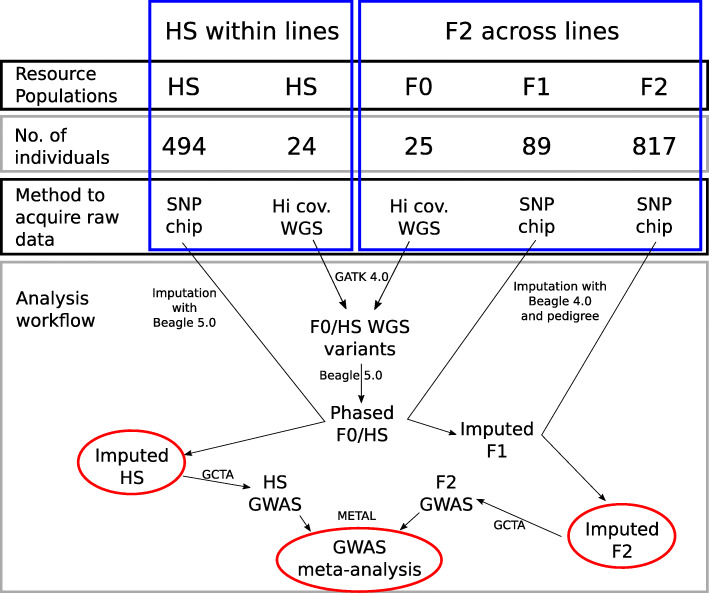
Analysis workflow from raw data to genome wide associations studies meta-analysis results. High coverage whole-genome sequencing (WGS) variants from an F2-cross and a half-sib (HS) population were used as a reference population for the imputation of low-density single nucleotide polymorphism (SNP) chip genotypes

Haplotype phasing and imputation of the various datasets was performed with Beagle 5.0. Beagle 4.0 was used when pedigree data was involved, since it is the latest version to support pedigree data. The phasing/imputation workflow is summarized in Fig. 4. Prior to imputation, SNP chip data was filtered excluding monomorphic and unplaced markers as well as markers on sex chromosomes and with a call rate of less than 95%. Variants from WGS data were called jointly and used as a reference population for the imputation of the HS chip data and the F1 chip data. Imputed F1 genotypes were merged with the WGS genotype data and used as a reference population for the imputation of the F2 chip level genotypes. To compute the imputation accuracy imputation of the HS population was repeated 4 times, leaving out 4 animals that were chip genotyped and sequenced. The imputed genotypes for each animal were extracted from the files and merged. The WGS genotypes of the same animals were extracted from the variant call file and correlation (coefficient of determination, R^2^) for each variant on GGA1 and GGA7 was calculated with an in house R script. In total, 24 of the HS animals were sequenced for the reference population. None of the F1 and F2 animals were sequenced.

### Genome wide association studies

GWAS were performed with the Genome-wide Complex Trait Analysis (GCTA) software version 1.92.3 beta3 using the imputed sequence level genotype data filtered for MAF (> 0.01). In order to correct for relatedness between animals and stratification [6], a random genetic term was included based on a genomic relationship matrix calculated only from SNP-chip-data and following a leave one chromosome out (LOCO) approach as described here [10]. Briefly, a model of the form ***y = Wα + Xβ + u + ϵ*** *is* fitted, where **y** is an **n × 1** vector of phenotypes **n** hens (a summary of the phenotype data is shown in Table 5). **W** is an **n × c** incidence matrix of fixed effects with **c** being the number of effects and **α** is a vector of corresponding coefficients including the mean. Further, the effects of line (HFP and LFP) and hatch were included in the analysis of the HS design, while only hatch was included for the F2 design. **X** is an **n × 1** vector of marker genotypes at the locus tested and **β** is the corresponding effect size; and **u** is a vector of random genetic effects, with $\boldsymbol{u}\sim N\left(0,{\boldsymbol{A}}^{-}{\sigma}_g^2\right)$, where ${\sigma}_g^2$ represents genetic variance and **A**^**−**^ is the genomic relationship matrix based on all SNP-chip markers except those on the chromosome currently analyzed. Finally, **ε** is a random residual term, with $\boldsymbol{\epsilon} \sim N\left(0,\boldsymbol{I}{\sigma}_{\epsilon}^2\right)$, where ${\sigma}_{\epsilon}^2$ represents the residual variance and **I** represents an identity matrix.

**Table 5:** Summary of phenotype values used in the genome wide analysis studies for the traits ‘feather pecks delivered’ (FPD) and the ‘posterior probability of a hen to belong to the extreme feather pecking subgroup’ (pEFP)

	F2	HS HFP	HS LFP
No. of animals	817	270	221
FPD average	13.91	10.35	1.50
FPD SD	26.19	16.69	1.94
FPD min	0	0	0
FPD max	198.33	115.50	15
pEFP average	0.28	0.31	0.20
pEFP SD	0.45	0.29	0.15
pEFP min	0	0.12	0.11
pEFP max	1	1	1

The variance explained by all markers on the SNP-chip was estimated using the same model without the SNP effect. In the F2 design, the variance explained by all significantly associated markers was estimated by including a second GRM based on the significant SNPs. As in the HS design only a single significant QTL was found with several adjacent markers, we partitioned the variance into a component explained by the SNP chip markers within a 5 Mb QTL window and the rest of the genome.

Meta-analysis of the GCTA output for each trait was performed with METAL version 1.1 using the sample size based approach with default settings [59].

### Variant effect prediction

The Ensembl Variant Effect Predictor release 98, which is part of the Ensembl advanced programming interface (API), was used for variant effect prediction with the following settings: --cache --species chicken --refseq --force_overwrite --variant_class --symbol --nearest symbol --sift b --tab.

### Functional analyses

Gene cluster analyses were conducted using the R package clusterProfiler [23] along with the chicken genome annotation org. Gg.eg.db [60] and using default settings in terms of gene set size and *p*-value correction. Protein interaction networks were computed with STRING v. 11 [61] with default settings.

## Supplementary information

**Additional file 1.** Coefficients of determination of imputation accuracy on GGA1 and GGA7 for the HS population.

**Additional file 2.** Variants (−log10(p) > 5) from all GWAS and meta-analysis performed with the traits FPD and pEFP.

**Additional file 3.** Manhattan plots of the GWAS performed with the F2 cross and the HS structure with the traits FPD and pEFP.

**Additional file 4.** Venn diagrams of associated genes from the GWAS and meta-analyses with the traits FPD and pEFP.

**Additional file 5.** List of genes concordant and discordant between the GWAS and meta-analyses with the traits FPD and pEFP.

**Additional file 6.** Results of the VEP of genes affected by variants (−log10(p) > 5) from the GWAS and meta-analyses with the traits FPD and pEFP.

**Additional file 7.** Results of the gene set enrichment analysis of genes affected by variants (−log10(p) > 5) from the GWAS meta-analyses with the traits FPD and pEFP with clusterProfiler.

**Additional file 8.** Protein interactions maps of genes affected by variants (−log10(p) > 5) from the GWAS with the traits FPD and pEFP.

## Data Availability

The datasets used and/or analysed during the current study are available from the corresponding author on reasonable request. The raw WGS data is available under BioProject ID PRJNA664592.

## Abbreviations

EFP: Extreme feather pecking
FP: Feather pecking
FPD: Feather pecks delivered
FPKM: Fragments per kilobase of exon model per million reads mapped
GWAS: Genome wide associations study/studies
HFP: High feather peckers
HS: Half-sib
LFP: Low feather peckers
nAchR: nicotinic acetylcholine receptors
ncRNA: non-coding RNA
NMDARs: N-methyl-d-aspartate receptors
pEFP: posterior probability of hens to belong to the extreme feather pecking subgroup
VEP: Variant effect prediction
WGS: Whole genome sequencing
