# Supplementary material for: Meta-analyses of genome wide association studies in lines of laying hens divergently selected for feather pecking using imputed sequence level genotypes
Source: BMC Genet. 2020 Oct 1;21:114. doi: 10.1186/s12863-020-00920-9 (PMC7528462; doi:10.1186/s12863-020-00920-9)
Supplement: Supplementary file 3 — Additional file 3. Manhattan plots of the GWAS performed with the F2 cross and the HS structure with the traits FPD and pEFP. [file 12863_2020_920_MOESM3_ESM.pdf]

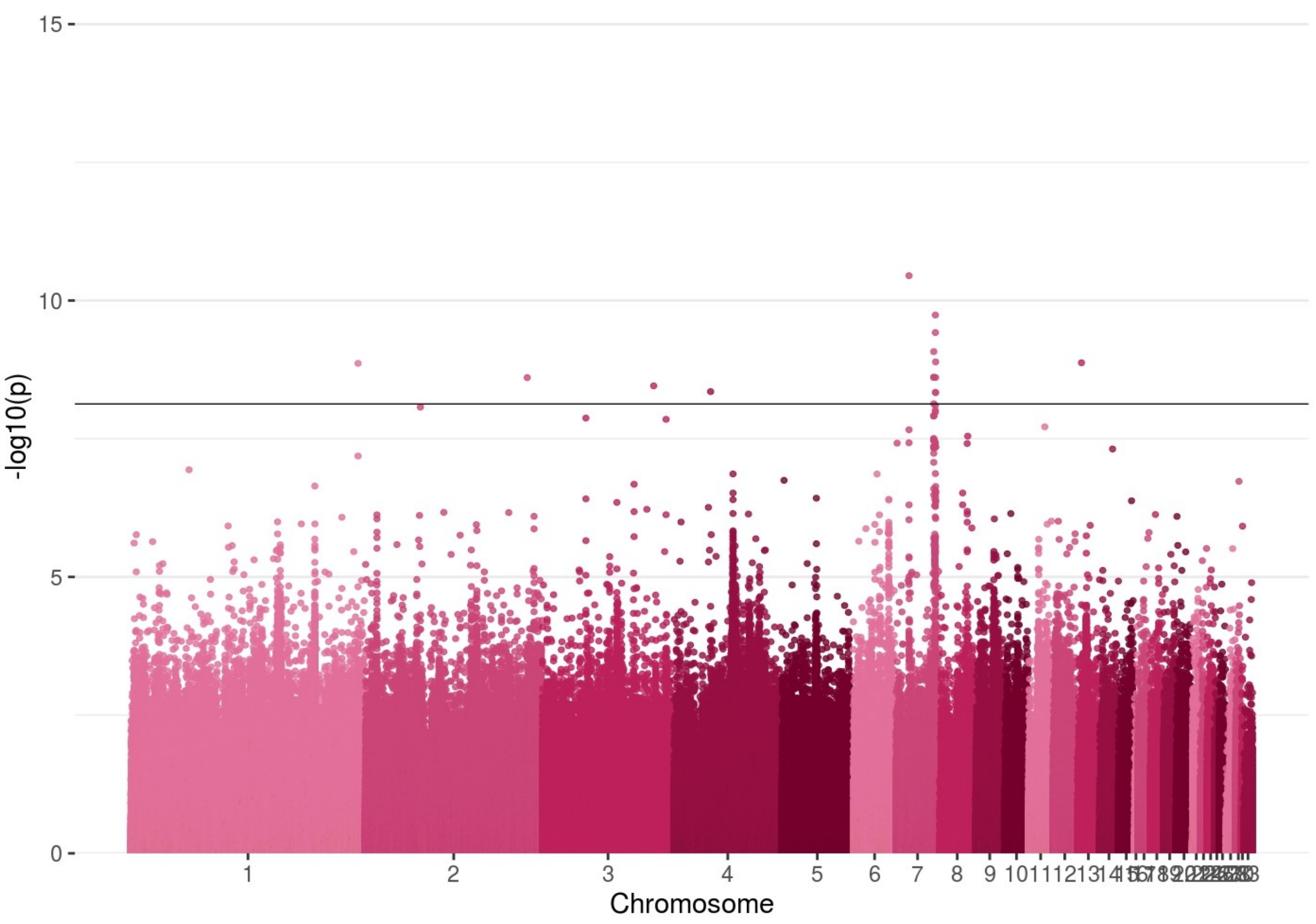

Manhattan plot of GWAS with the F2 population performed with the trait FPD

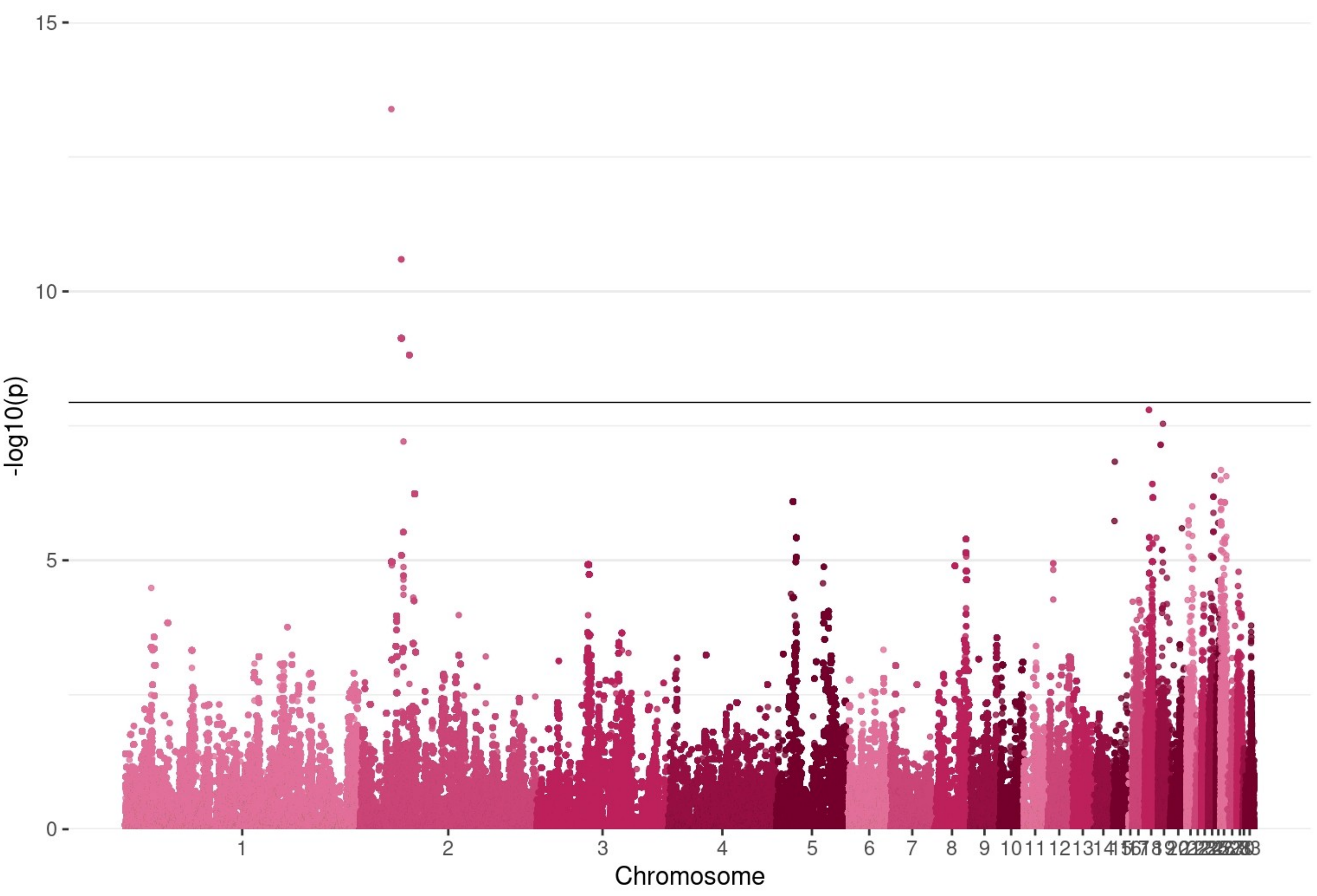

Manhattan plot of GWAS with the HS population performed with the trait FPD

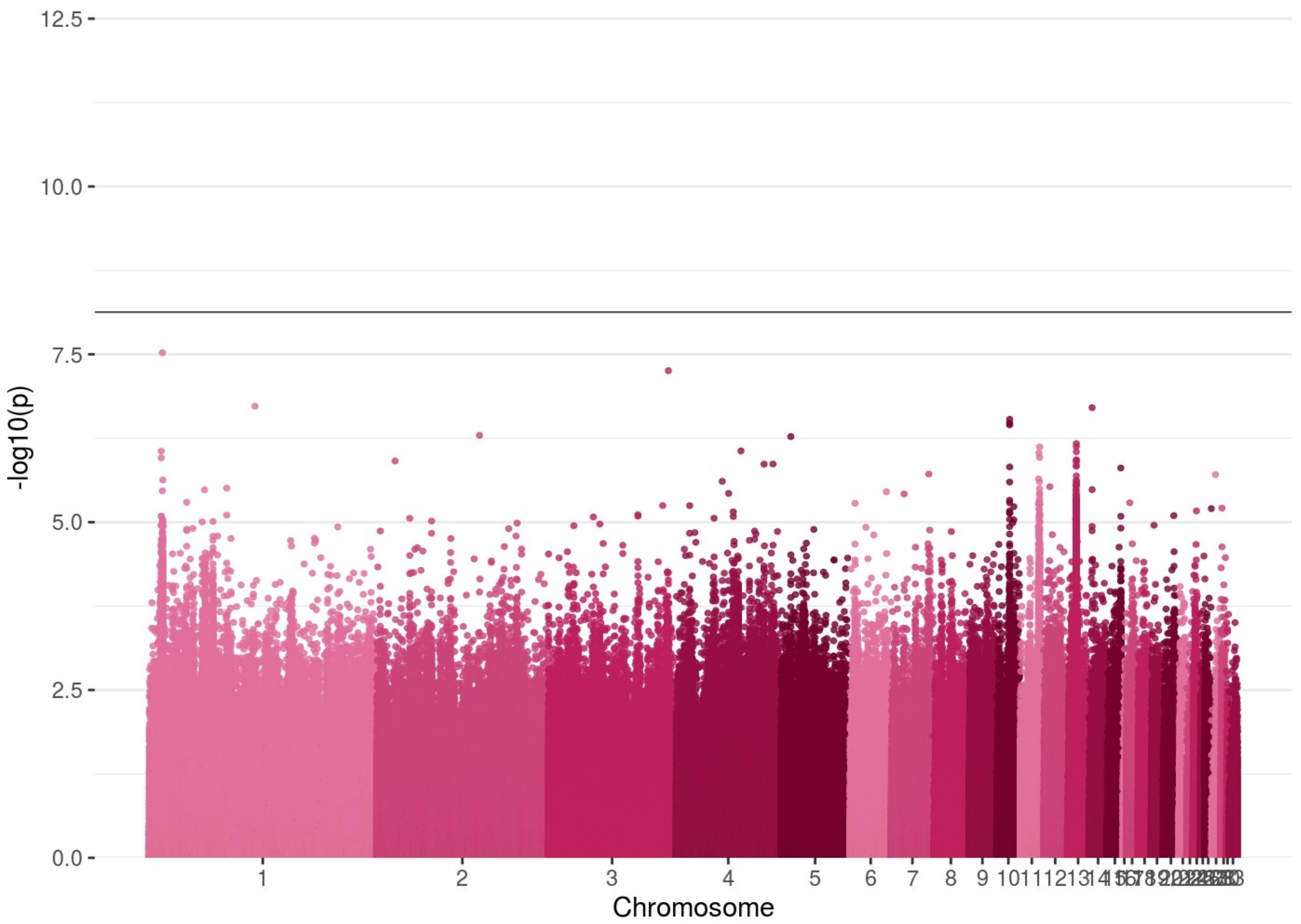

Manhattan plot of GWAS with the F2 population performed with the trait pEFP

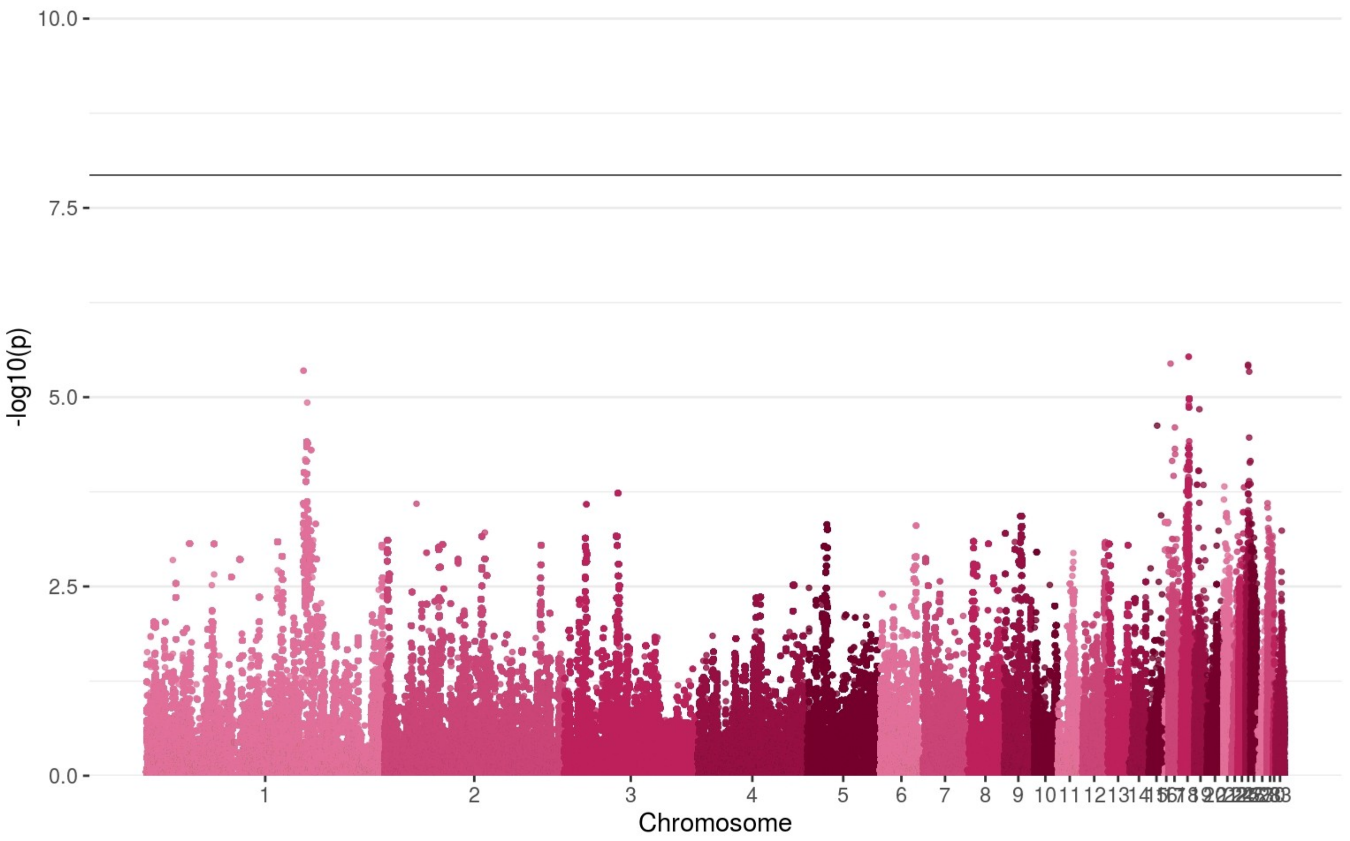

Manhattan plot of GWAS with the HS population performed with the trait pEFP
