# Supplementary material for: Meta-analyses of genome wide association studies in lines of laying hens divergently selected for feather pecking using imputed sequence level genotypes
Source: BMC Genet. 2020 Oct 1;21:114. doi: 10.1186/s12863-020-00920-9 (PMC7528462; doi:10.1186/s12863-020-00920-9)

Venn diagrams of associated genes from the GWAS and meta-analyses with the traits FPD and pEFP

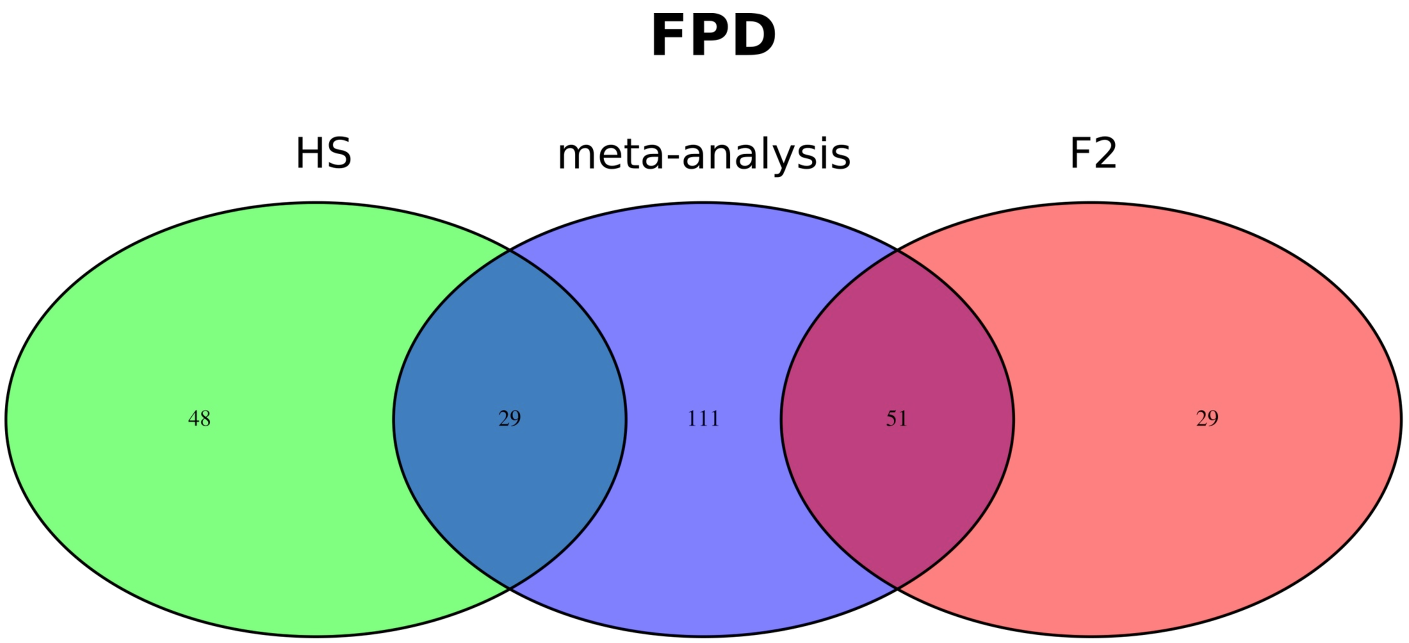

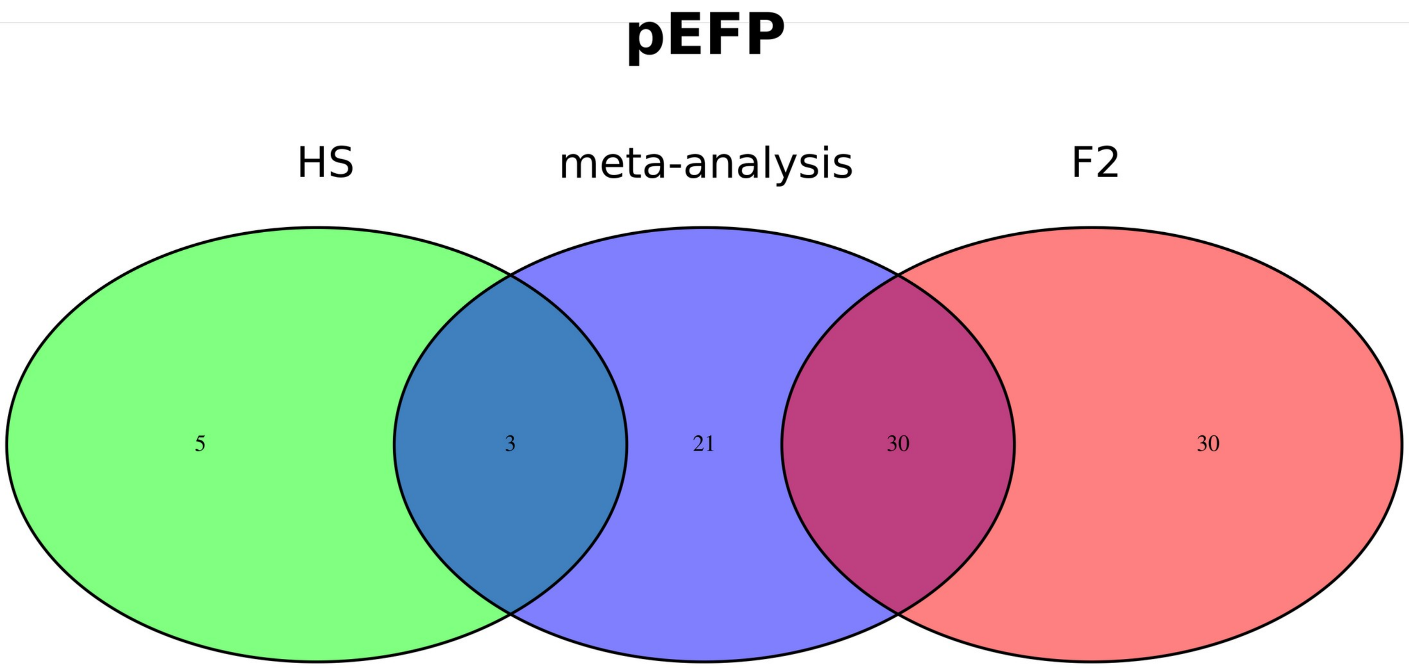

Supplement: Supplementary file 4 — Additional file 4. Venn diagrams of associated genes from the GWAS and meta-analyses with the traits FPD and pEFP. [file 12863_2020_920_MOESM4_ESM.pdf]
