# Supplementary material for: Meta-analyses of genome wide association studies in lines of laying hens divergently selected for feather pecking using imputed sequence level genotypes
Source: BMC Genet. 2020 Oct 1;21:114. doi: 10.1186/s12863-020-00920-9 (PMC7528462; doi:10.1186/s12863-020-00920-9)

Protein interaction network analysis of genes associated with variants with -log10(p) > 5 for the phenotype FPD in the F2 resource population

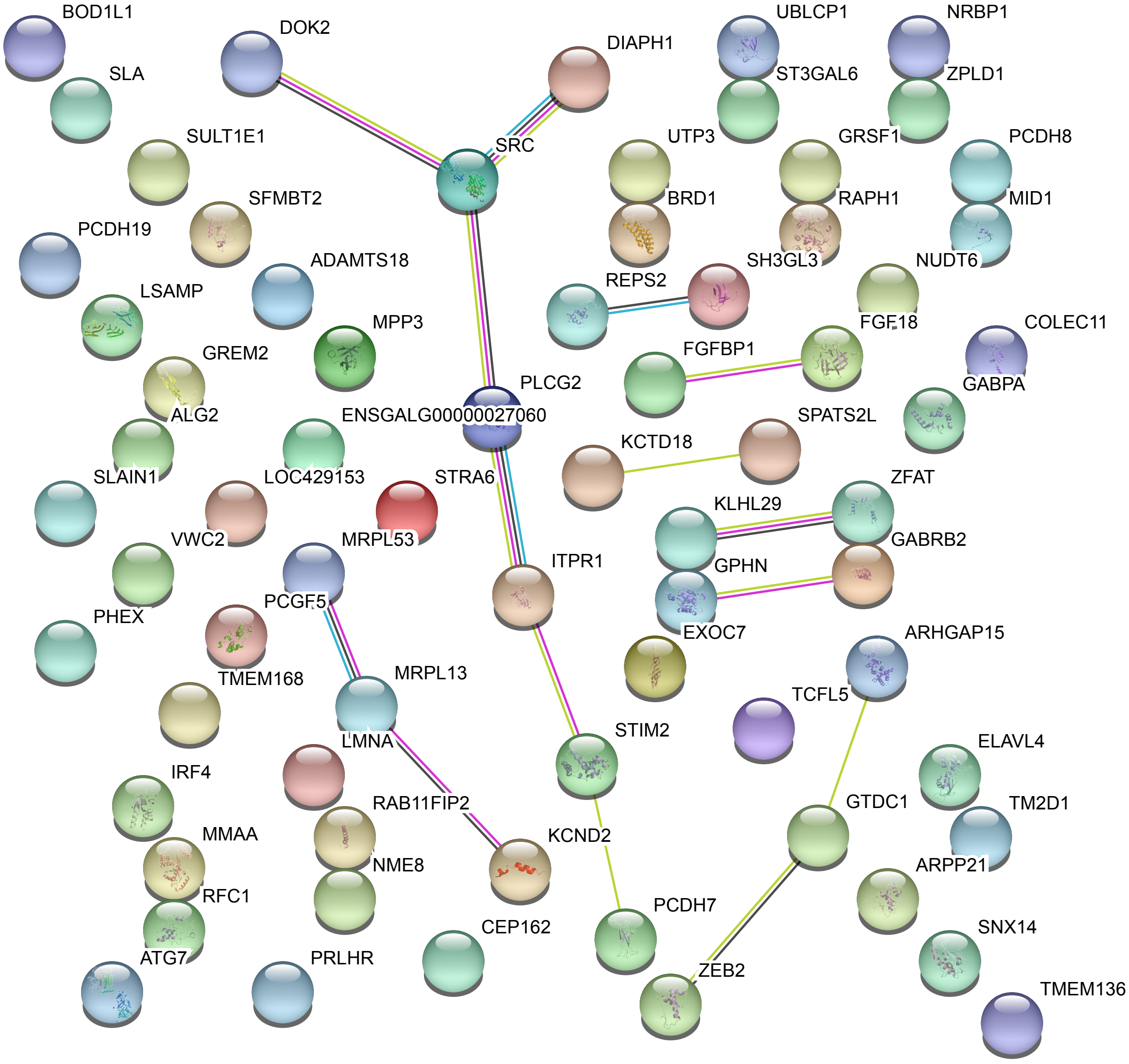

Protein interaction network analysis of genes associated with variants with -log10(p) > 5 for the phenotype FPD in the HS resource population

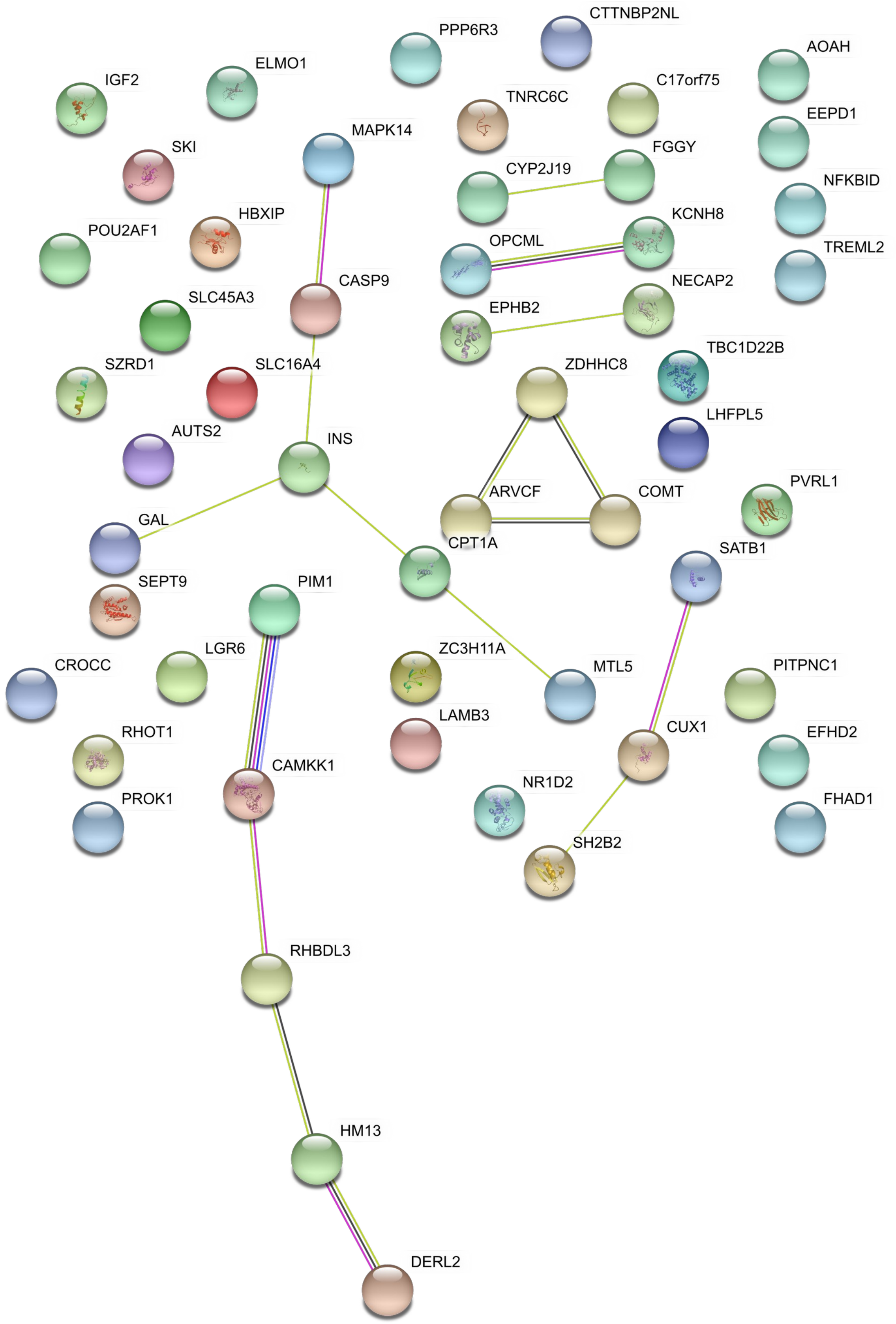

Supplement: Supplementary file 8 — Additional file 8. Protein interactions maps of genes affected by variants (−log10(p) > 5) from the GWAS with the traits FPD and pEFP. [file 12863_2020_920_MOESM8_ESM.pdf]
